# Bisection of a Female Foetus: A Forensic Insight Into the Core Conclusions and Residual Constraints in Medicolegal Evaluation

**DOI:** 10.7759/cureus.107131

**Published:** 2026-04-15

**Authors:** Vimal Kanna G, Archit M Modi

**Affiliations:** 1 Forensic Medicine and Toxicology, Sumandeep Vidyapeeth (Deemed to be University), Vadodara, IND

**Keywords:** dismemberment, female foeticide, foetal viability, infanticide, live birth, postmortem scavenging, taphonomy

## Abstract

Forensic investigations in newborn deaths pose significant methodological and diagnostic challenges due to the fragility of the subject and subtlety of the postmortem findings. The investigative burden is further augmented when the remains are mutilated, and vital organs are absent owing to attempts at concealing the birth. In order to establish *corpus delicti* in such cases, identification of the foetus, assessing its viability and determining whether it was a live birth or not are imperative. The determination of live birth is particularly elusive because the conventional methods of estimating live birth analyse organs of the upper torso or intact viscera exclusively. In situations when only the lower torso is available and where viscera have been destroyed by scavenging activity or putrefaction, estimating live birth is inconceivable. Scholarly works on this topic are also notably sparse, thus leaving a significant lacuna in the existing works of literature.

We report a case of a bisected female foetus recovered from an open vegetation, of which only the lower torso and lower extremities were found, with an anthropogenic sharp-force transection at the thoracic level superimposed by postmortem faunal scavenging. Gender and period of viability could be established from the available evidence, but the absence of the upper half of the body, including all vital organs, along with putrefactive changes, posed a serious constraint in evaluating live birth. The unavailability of the usual determinants of livebirth hindered the unravelling of the complete truth. Hence, there is a need for further scientific exploration to overcome these limitations. Future studies should fruitfully explore this issue, as further inquiry into this area is warranted for a more comprehensive understanding. Documenting these extreme methods of disposal is crucial to addressing the tragic reality of gender-biased foeticide.

## Introduction

Infanticide remains a major social problem in developing countries, mainly influenced by socio-economic pressures and the age-old gender bias. Due to the societal and patriarchal norms, cases of female foeticide, infanticide, and concealment of birth are a frequent occurrence in India [1,2]. The most common forms of infanticide are suffocation and strangulation, whereas blunt force injuries and poisoning are some rarer methods [3]. While most such cases do not involve dismemberment, the discovery of partial or severely mutilated remains gives rise to a complex diagnostic dilemma with serious medico-legal consequences. Disposal of unwanted neonates usually involves drowning, burning of the corpse, or abandonment in secluded areas [4]. The legal punishment in India can largely vary from the Bharatiya Nyaya Sanhita (BNS) 91 in cases of killing an infant to BNS 94 if the infant is not alive at birth, where the crime will only amount to concealment of birth [5]. Thus, it is absolutely vital to understand the details of the case, as it can drastically change the magnitude of the action of the law. 

To complete a forensic investigation, multiple queries have to be solved to establish *corpus delicti*. It is important to ascertain whether the body was of a viable infant, if the neonate was stillborn or born alive, and if born alive, for how long. Apart from these, the cause of death has to be established as well. The conclusions of these investigations are legal prerequisites to establishing charges of crime. Cases of partial remains give rise to even more challenges, such as differentiating between anthropogenic mutilation and taphonomic artefacts. In the absence of vital organs, such as the lungs and the brain, most standard tests to answer the above questions become invalid. 

We report a rare case of a viable, preterm female foetus, of which only the bisected lower half was recovered. This case features the dual mechanism of trauma, in which deliberate sharp force transection was superimposed by post-mortem animal scavenging. We discuss the forensic methodology used for the examination in this case and raise a scientific inquiry on the approaches of differentiating between live birth and stillbirth in unorthodox cases of partial, fragmented remains when routine procedures are impracticable.

## Case presentation

**Figure 1:**
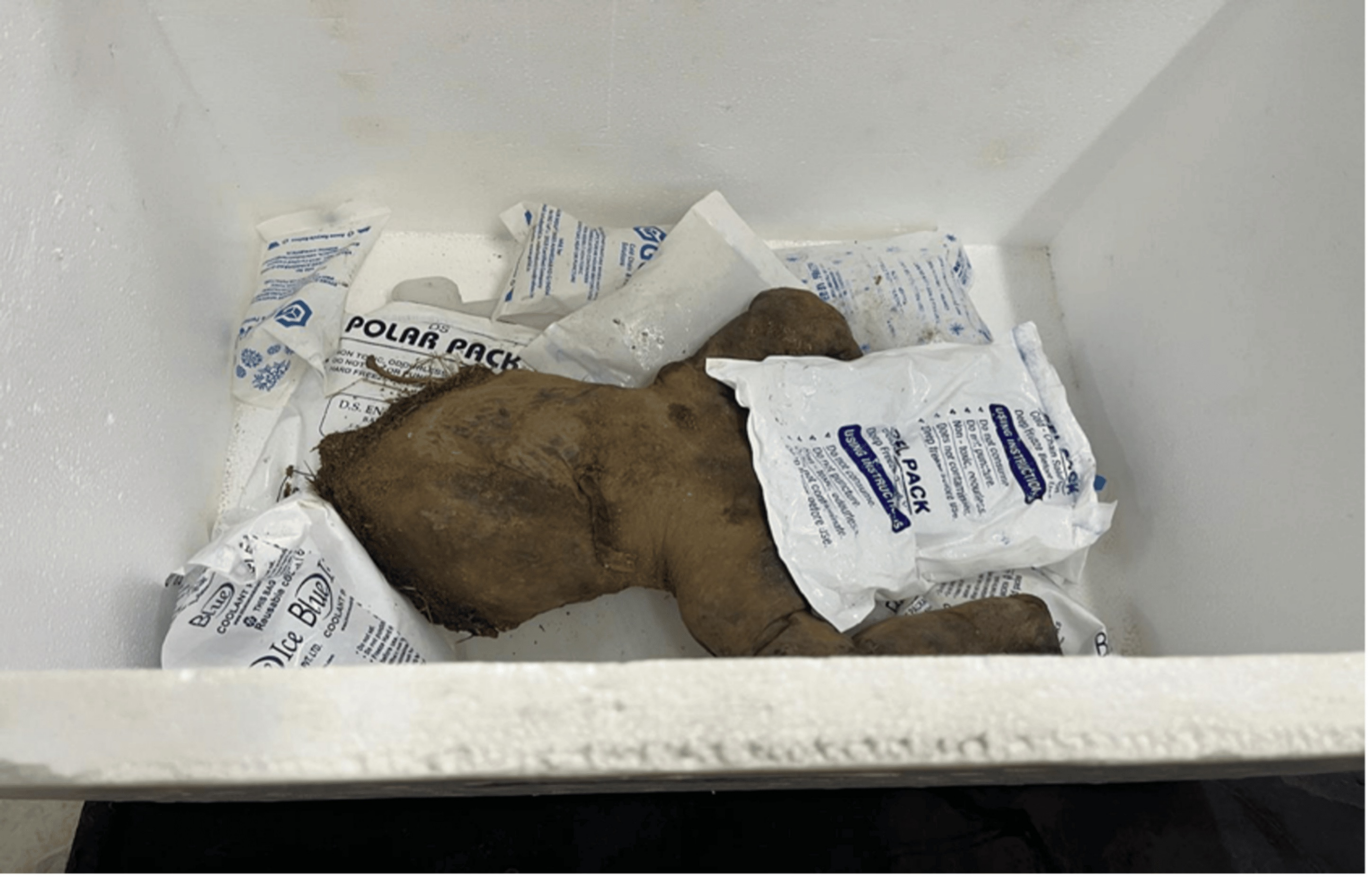
The recovered partial foetus showing dismemberment

The lower bisected half of an unidentified foetus was recovered from open vegetation and brought for forensic examination. The history provided by the investigating officer stated that the remains were discovered with no clothing or placenta found at the crime scene (Figure 1).

Autopsy findings

External Examination

The head, both upper limbs, and the thorax, except the sternal area, were missing. Soft tissue from the left gluteal region, and all the toes of the right foot were missing. The abdominal cavity was exposed at the upper aspect, and the intestines and soft tissues were protruding out of it. The umbilical cord was 8 cm long with irregular margins at the end.

**Figure 2:**
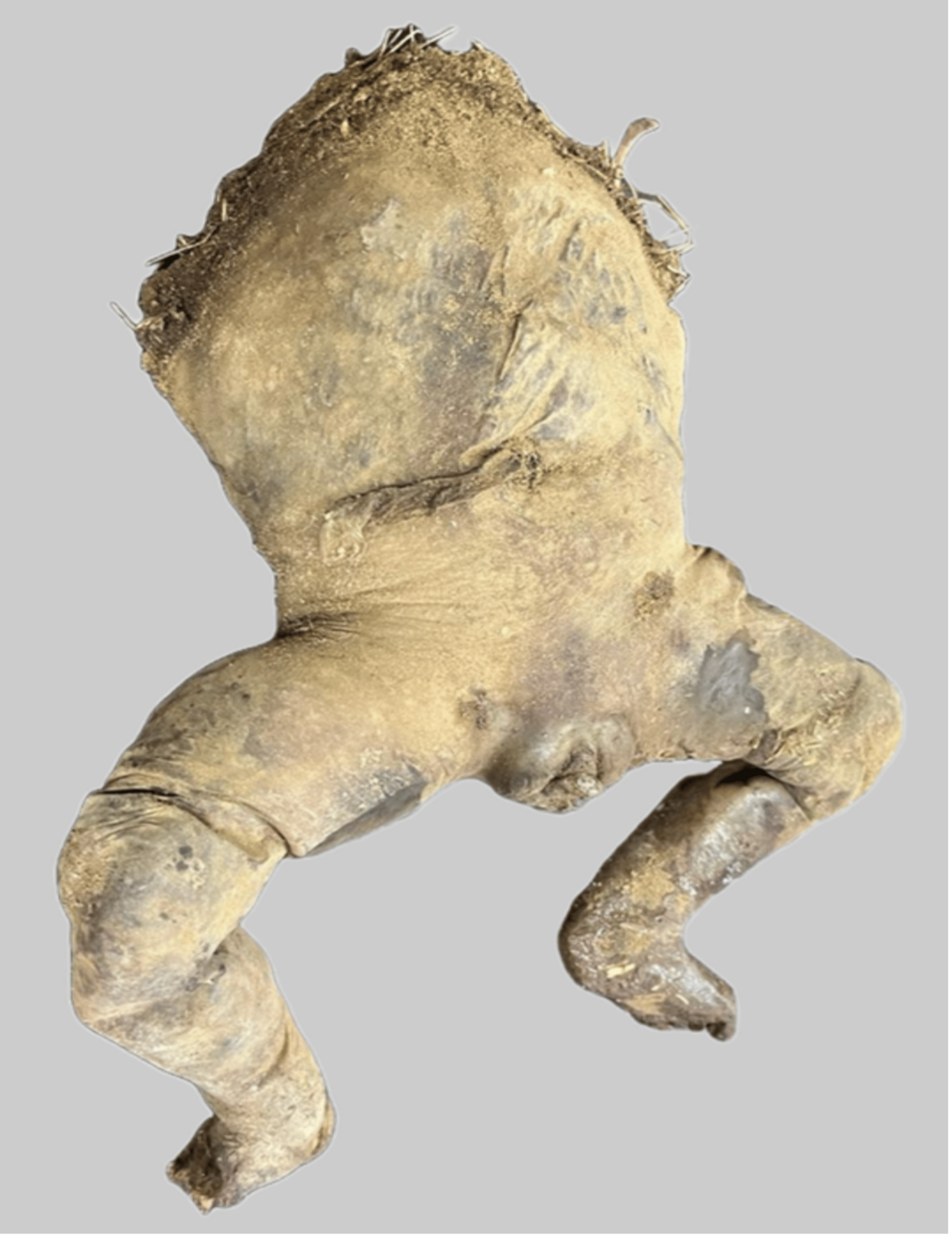
The anterior aspect, with the torn umbilical cord

**Figure 3:**
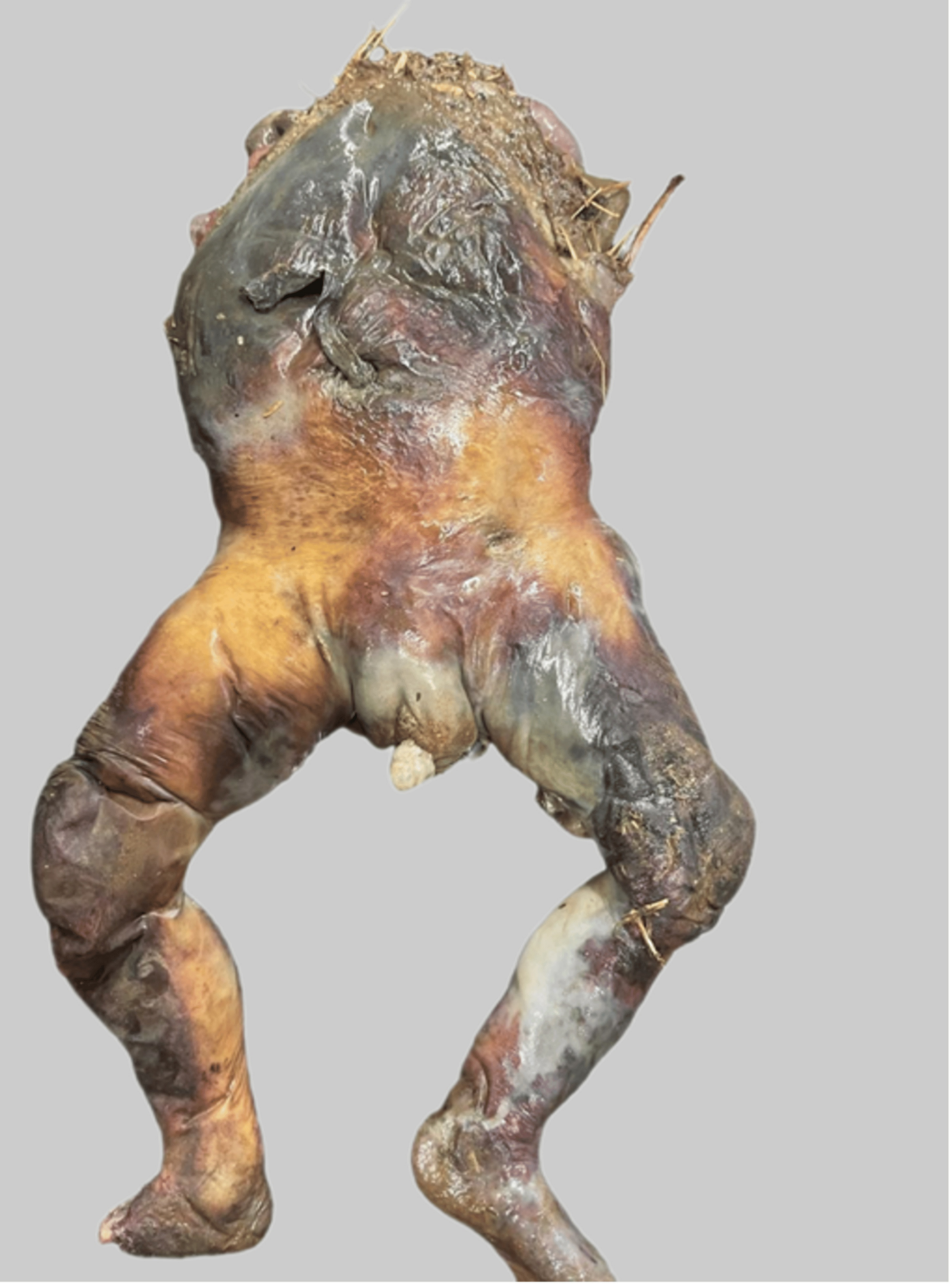
The posterior aspect showing marked putrefaction

The external genitalia of the foetus were identified as female sex. The total length of the available corpus was 27 cm, and the weight was 540 grams. The body was foul-smelling and in a state of putrefaction (Figures 2, 3).

Injuries/Taphonomy

**Figure 4:**
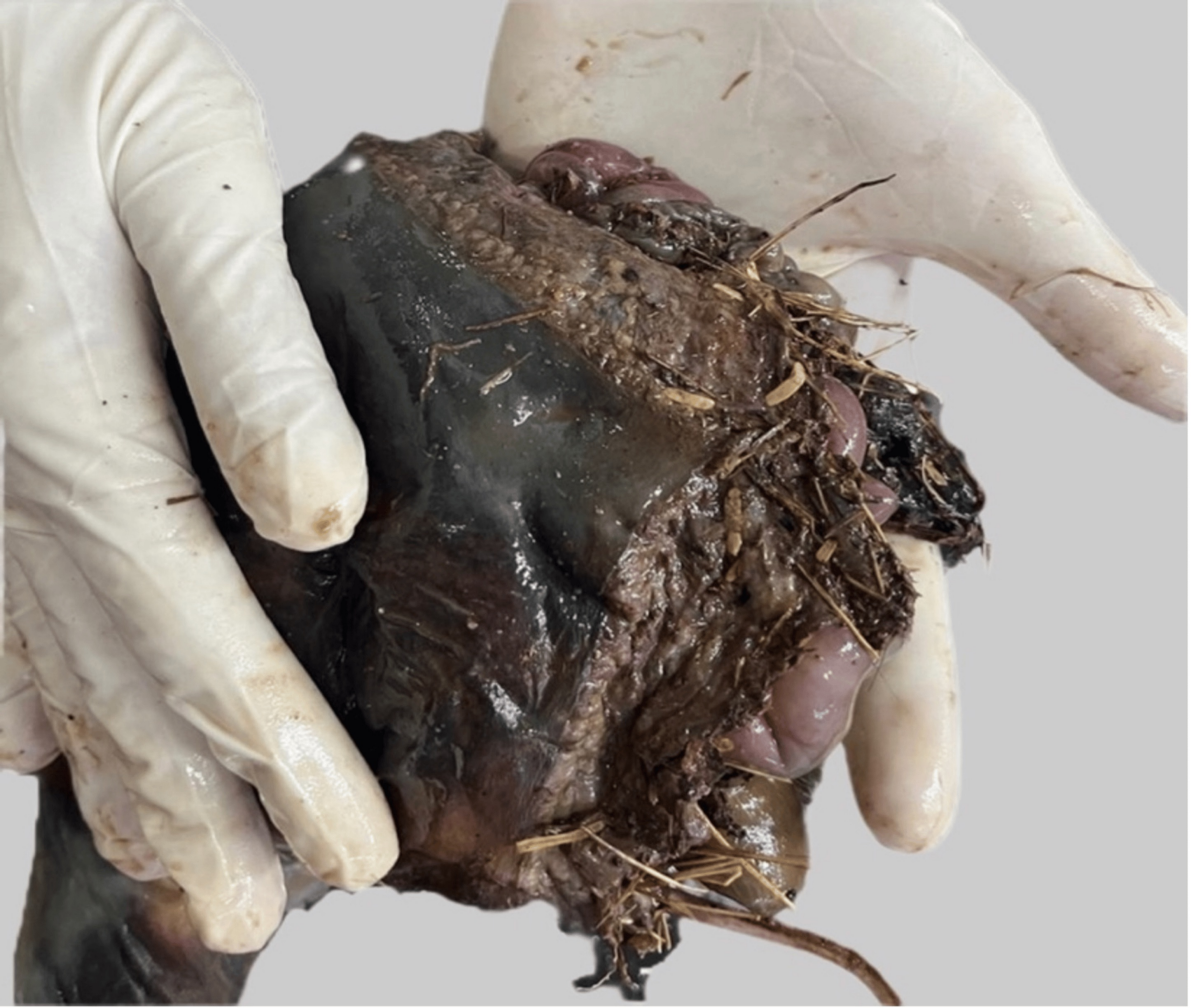
Planar transverse transection of the trunk; regular, linear and sharp margins are seen

Perimortem trauma (the human cut): A complete, planar transverse transection of the trunk was seen at the thoracic level with regular, linear and sharp margins (Figure 4).

**Figure 5:**
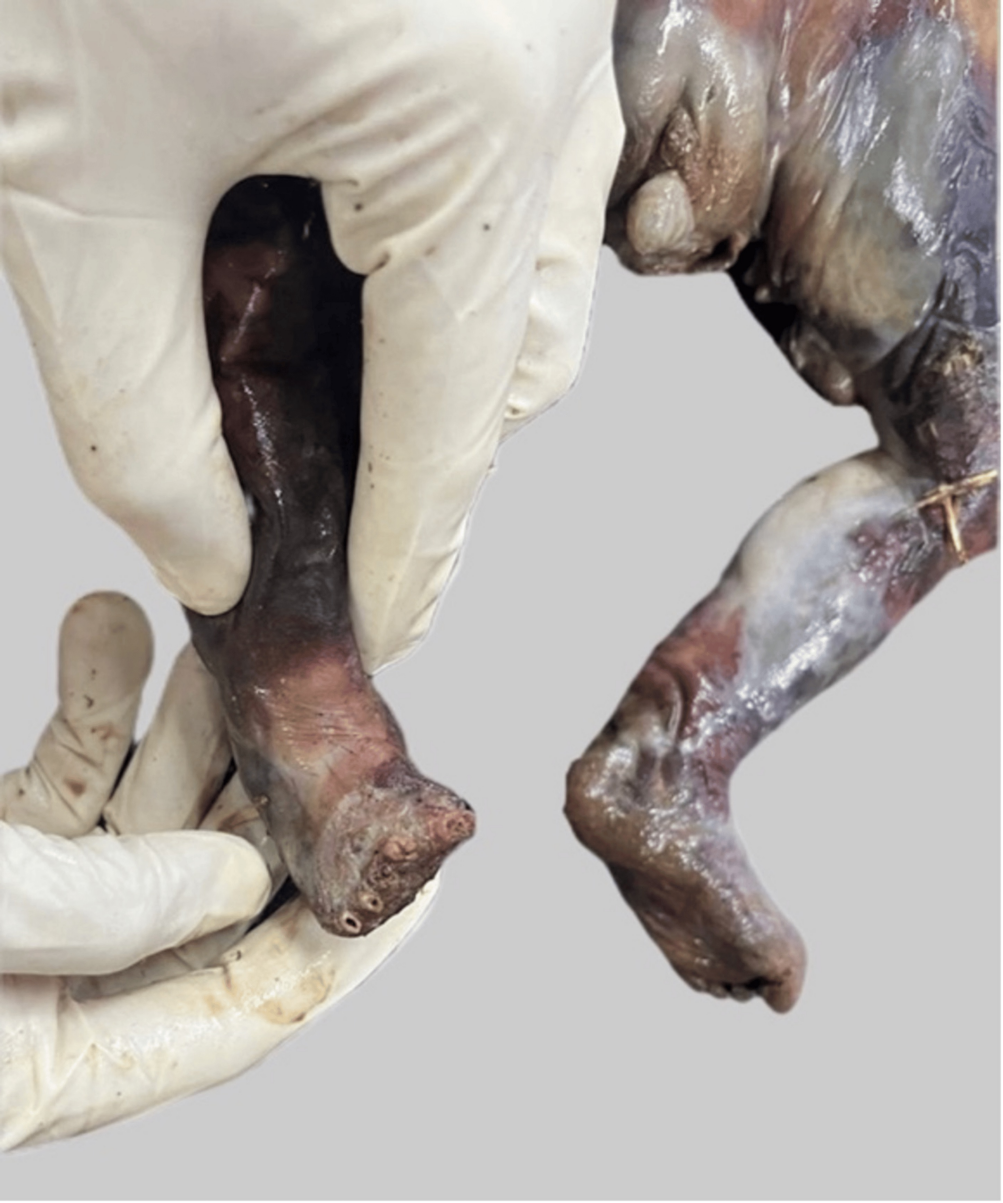
Missing toes of the right foot; serrated margins and nibbling marks are noted which are positive of animal scavenging.

Postmortem trauma (animal scavenging): Irregular, ragged defects were seen on the right lower limb and left gluteal region. The margins are serrated with crater-like tissue loss, which is consistent with faunal scavenging activity (Figure 5).

Internal Examination

**Figure 6:**
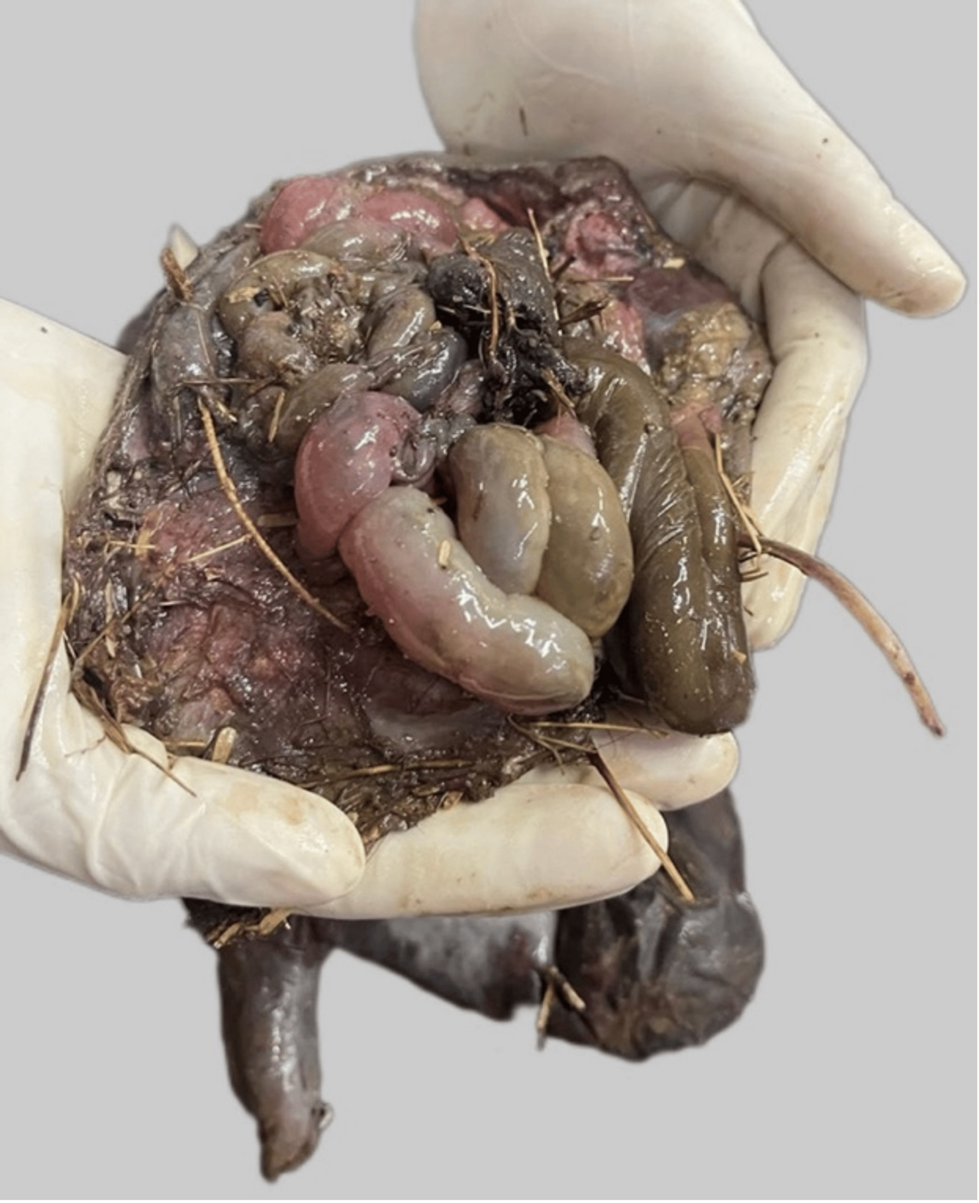
The superior view, exposing the visible protruding intestines and remaining contents of the abdominal cavity.

Meconium was present in the entire large intestine. The remaining available organs, i.e. kidneys, bladder, and uterus, showed advanced autolytic changes and were decomposing. None of the other organs were found (Figure 6).

Osteology

**Figure 7:**
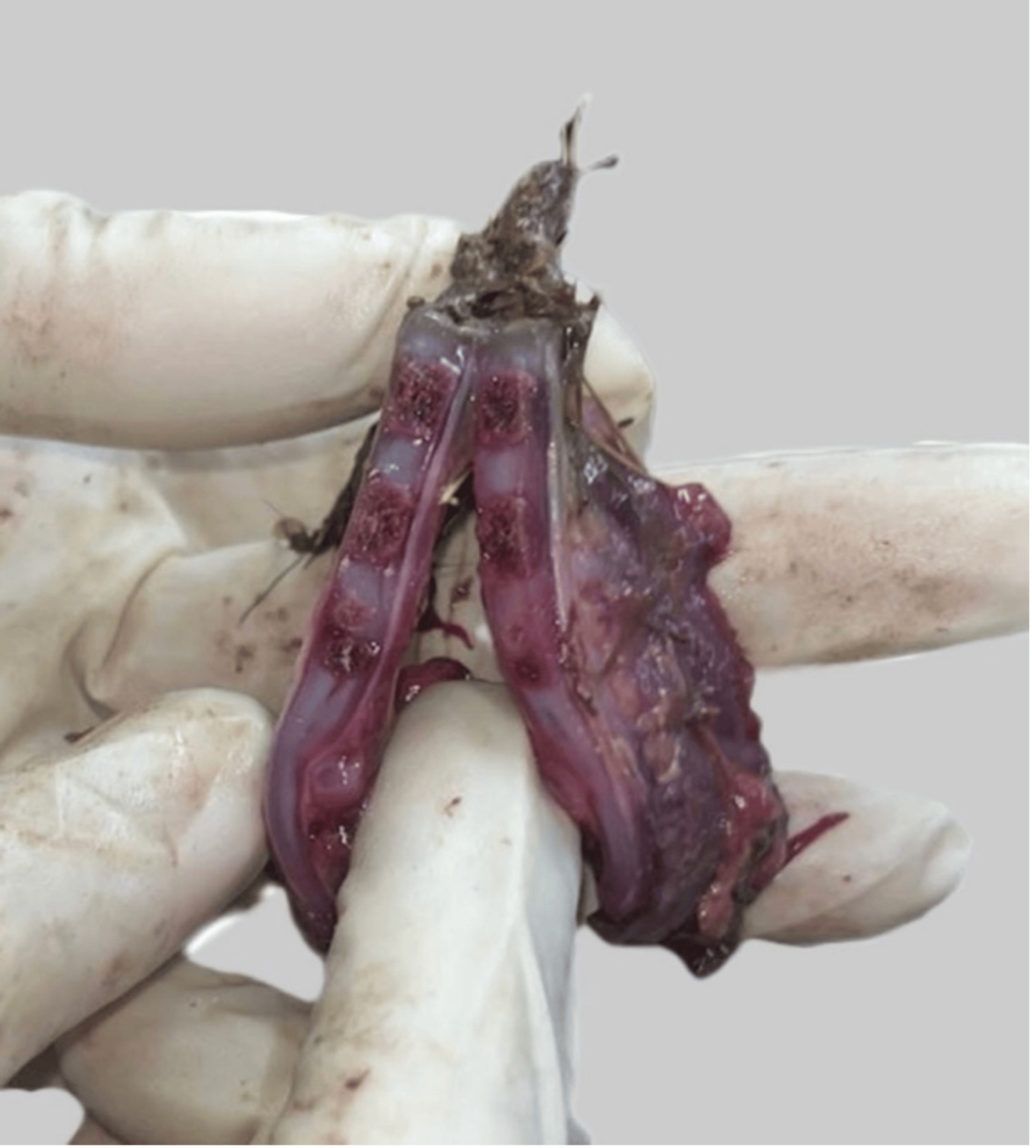
The ossification centres seen in the cut cross-section of the sternum

**Figure 8:**
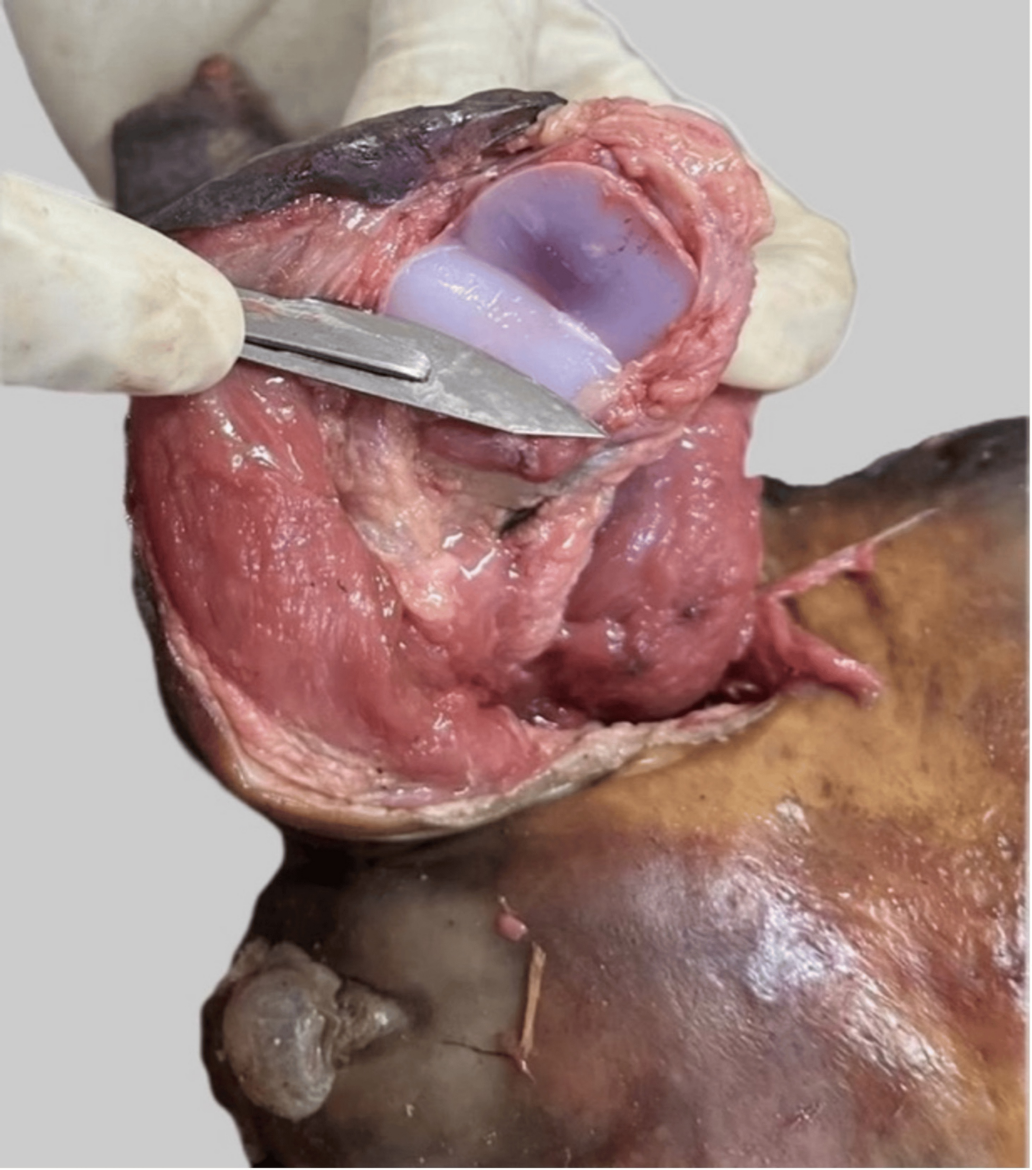
No ossification seen at the distal femoral epiphysis

**Figure 9:**
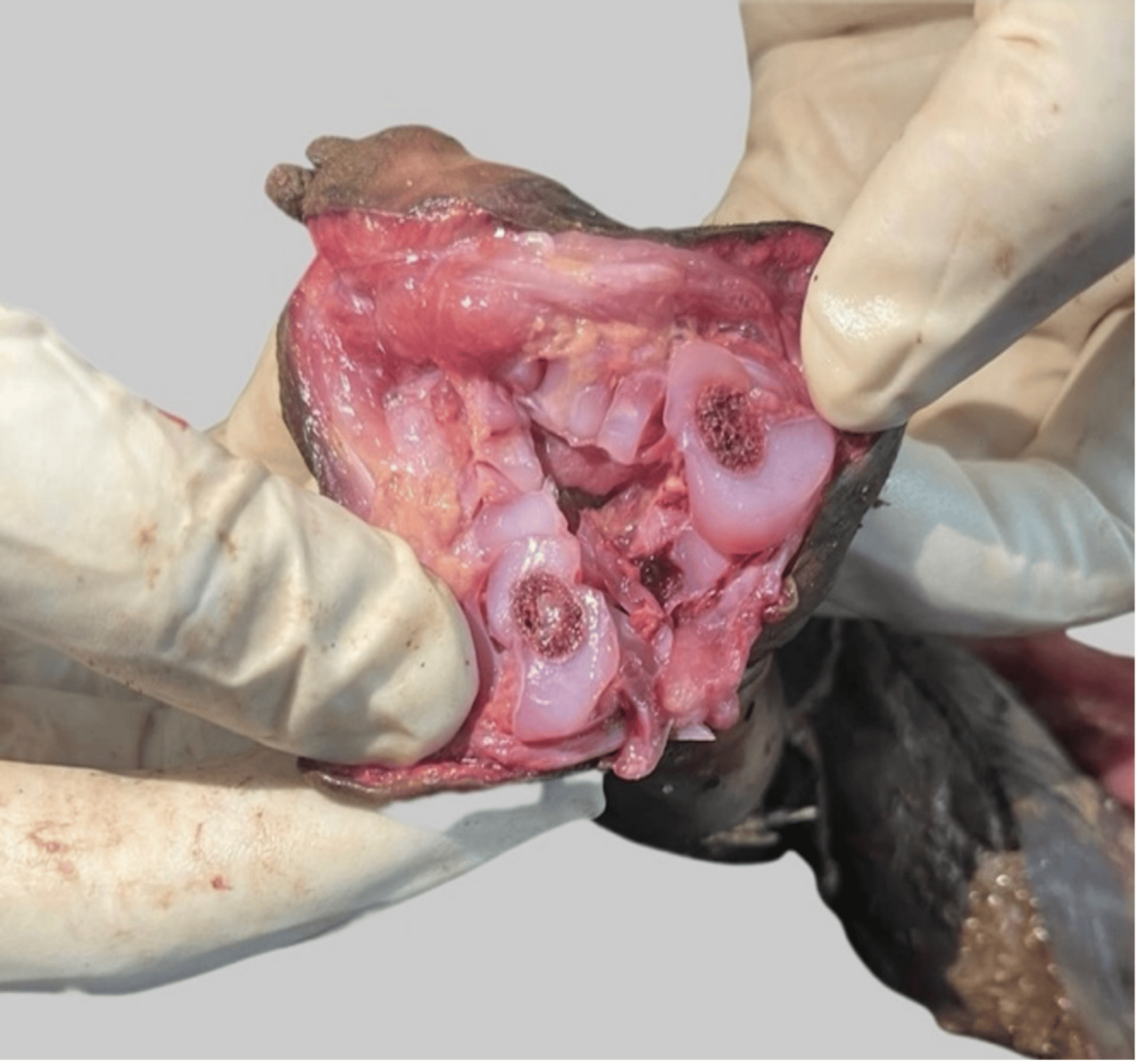
Ossification centre at the calcaneum

**Figure 10:**
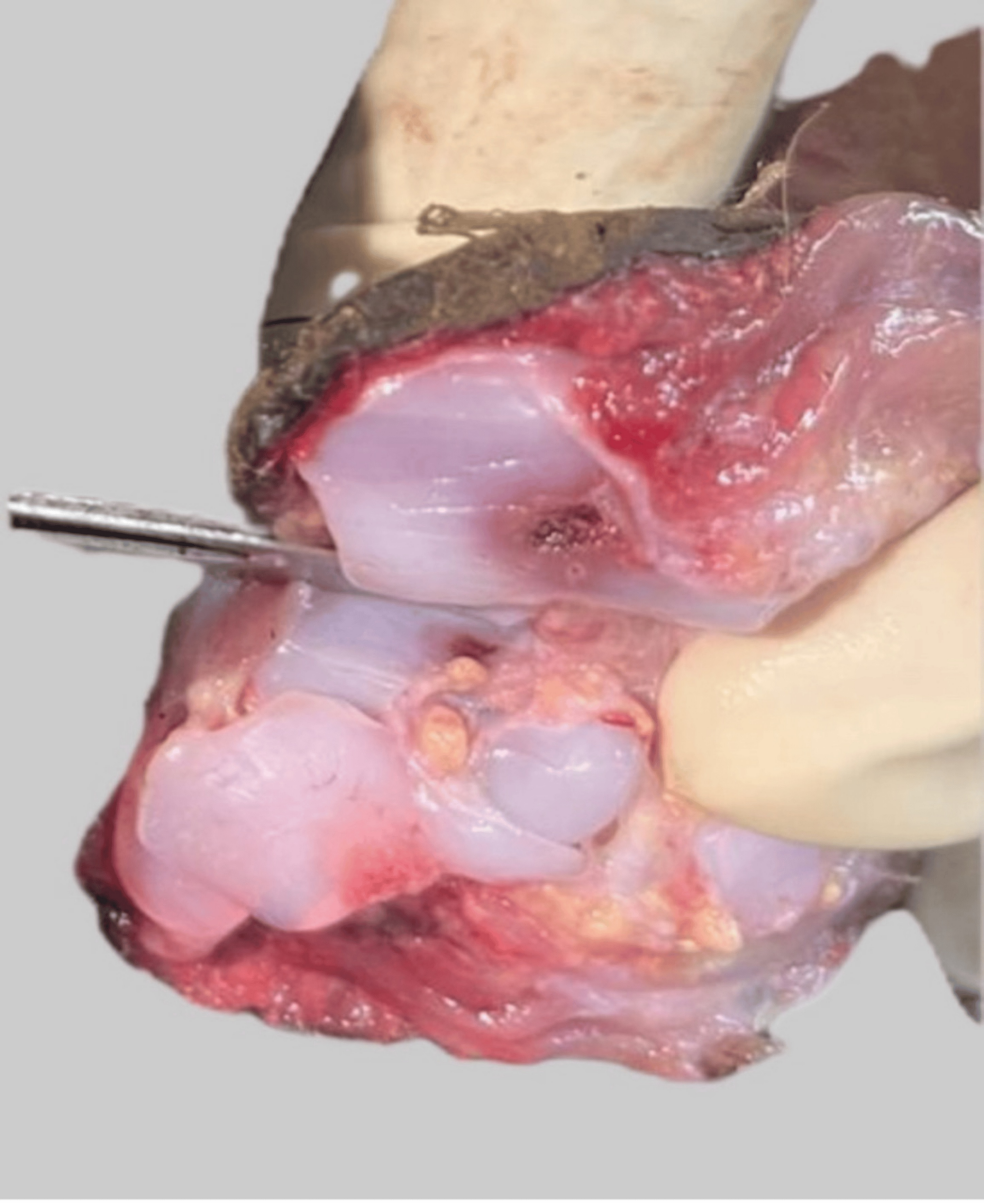
Ossification centre at the talus

In the sternum, ossification centres were present in the manubrium, and first, second, and third segments of the body of the sternum. Ossification centres were also present in the calcaneus and talus, but were absent in the distal femoral epiphysis (Figures 7-10).

## Discussion

The discovery of partial remains in open vegetation raises the need for distinction between human and faunal activity for the cause and manner of death. In this case, there was a clear-cut marker of human dismemberment based on the geometric, planar separation of the body. This clearly indicates an intentional human act of mutilation, likely to facilitate the disposal of a criminal activity. The serrated margins on the right lower extremity and left gluteal tissue were a result of taphonomic changes due to animal scavenging.

Ossification centres present in the calcaneum (appearing at five to six months) (Figure 9) and the talus (appearing at seven months) (Figure 10), along with the third sternebra (appearing at seven months) (Figure 7), confirmed the gestation of a minimum of 28 weeks. The absence of the distal femoral epiphysis (Figure 8) placed a ceiling on the gestational age of 36 weeks. Integrating the osteological findings with visceral findings like the presence of meconium throughout the large intestine approximated the age of gestation between 34 and 36 weeks. This was a vital result, as although the foetus was preterm, it was biologically and legally viable. 

The dilemma between live birth and stillbirth could not be resolved. Currently, the standard methodology for determining live birth is limited. It encompasses various determinants that assess either probable or confirmed live birth, and which are mostly useful when specific parts of the body are available in a preserved condition. These determinants include assessing the lungs based on their morphology and histology, the lung flotation test/hydrostatic test and the Breslau Second Life test based on the external examination of the stomach and intestines. The diaphragm, middle ear, umbilical cord and placenta also play a notable role in the identification of probable livebirth. However, several studies have shown that it is scientifically invalid for such determinants to be the sole factor to make a decision, as elaborated by Sieswerda-Hoogendoorn T et al. [6]. Boreadis AG et al. [7] have also stated the unreliability of the Breslau Second Life test in determining live birth. Seldom are internal contents of the stomach and intestines, like the presence of milk or food, found that could be conclusive of a live birth. 

Newer studies present new modalities, such as the use of postmortem imaging (CT and MRI), as corroborative evidence, as has been described by Michiue T et al. [8]. Studies such as those by Neri M et al. [9] and Bonelli A et al. [10] highlight the use of microscopic examination of the umbilical cord in differentiating stillbirth and livebirth. Pathological changes seen in tissues are also strong evidence of a possible live birth.

In our case, since the upper half of the foetus, including all vital organs, was missing completely, and the available foetal part had also undergone advanced putrefactive changes, this part of the examination was inconceivable. However, there is a pressing need to explore and establish standard methods to overcome such limitations, as many cases of infanticides are found disposed of and in a putrefied state, proving it to be a global problem for all forensic pathologists, as a major part of their examination can be rendered inconclusive. 

## Conclusions

In this case, the proper forensic analysis of the available evidence by leveraging standard assessment techniques and the available literature, the diagnostic process provided clear, evidence-based results regarding the viability of the foetus, its gender and the manner of death. The availability of the uterus and the external genitalia confirmed the female gender of the foetus. The active method of violence raises a strong suspicion of gender-biased foeticide. Taking into account the available ossification centres, the age of the foetus was estimated between 34 and 36 weeks, which clearly indicated the foetus was viable. Thus, the forensic findings and manner of death almost successfully established the corpus delicti of the crime. This case underscores that even in cases of partial or fragmented remains, the osteological markers, wound analysis and understanding of internal contents remain significant to answer the legal and forensic questions raised during investigations. 

However, the critical question of whether the foetus was born alive or not remains unanswered in this case. The lack of sufficient peer-reviewed research and literature in such unusual complex scenarios makes it a daunting task for the medico-legal experts. The conventional methods of evaluating live birth are all solely dependent on the analysis of organs of the upper torso and atypical presentations such as in this case becomes an intricate conundrum. This serves as a pivotal restraint in establishing the facts of the crime and adversely hampers the appropriate judgment to be served. This case serves as a classic example that over-reliance on conventional methods can lead to a diagnostic stalemate, and hence, there is a pressing need to do more novel research so that we can bridge the knowledge deficit and develop more reliable protocols for evaluating and predicting live births when standard evidence falls short. Ultimately, overcoming the diagnostic limitations of partial remains is not just an academic exercise but also a legal necessity.
